# Blockade of Rostral Ventrolateral Medulla Apelin Receptors Does Not Attenuate Arterial Pressure in SHR and _L_-NAME-Induced Hypertensive Rats

**DOI:** 10.3389/fphys.2018.01488

**Published:** 2018-10-24

**Authors:** Philip R. Griffiths, Stephen J. Lolait, Louise E. Pearce, Fiona D. McBryde, Julian F. R. Paton, Anne-Marie O’Carroll

**Affiliations:** ^1^Laboratories for Integrative Neuroscience and Endocrinology, Bristol Medical School, University of Bristol, Bristol, United Kingdom; ^2^Department of Physiology, Faculty of Medical and Health Sciences, University of Auckland, Auckland, New Zealand; ^3^School of Physiology, Pharmacology and Neuroscience, University of Bristol, Bristol, United Kingdom

**Keywords:** apelin receptor, apelin, blood pressure, rostral ventrolateral medulla, hypertensive rats

## Abstract

Dysfunction of the apelinergic system, comprised of the neuropeptide apelin mediating its effects via the G protein-coupled apelin receptor (APJ), may underlie the onset of cardiovascular disease such as hypertension. Apelin expression is increased in the rostral ventrolateral medulla (RVLM) in spontaneously hypertensive rats (SHRs) compared to Wistar-Kyoto (WKY) normotensive rats, however, evidence that the apelinergic system chronically influences mean arterial blood pressure (MABP) under pathophysiological conditions remains to be established. In this study we investigated, in conscious unrestrained rats, whether APJ contributes to MABP and sympathetic vasomotor tone in the progression of two models of hypertension – SHR and _L_-NAME-treated rats – and whether APJ contributes to the development of hypertension in pre-hypertensive SHR. In SHR we showed that APJ gene (*aplnr*) expression was elevated in the RVLM, and there was a greater MABP increase following microinjection of [Pyr^1^]apelin-13 to the RVLM of SHR compared to WKY rats. Bilateral microinjection of a lentiviral APJ-specific-shRNA construct into the RVLM of WKY, SHR, and _L_-NAME-treated rats, chronically implanted with radiotelemeters to measure MABP, decreased *aplnr* expression in the RVLM and abolished acute [Pyr^1^]apelin-13-induced increases in MABP. However, chronic knockdown of *aplnr* in the RVLM did not affect MABP in either SHR or _L_-NAME-treated rats. Moreover, knockdown of *aplnr* in the RVLM of prehypertensive SHR did not protect against the development of hypertension. These results show that endogenous apelin, acting via APJ, is not involved in the genesis or maintenance of hypertension in either animal model used in this study.

## Introduction

The brainstem plays a vital role in blood pressure (BP) regulation through an intricate signaling network that modulates sympathetic nerve activity (SNA) and secretes hormones into the circulation to maintain a homeostatic BP (Fisher and Paton, 2012). Increasing evidence supports a role for the neuropeptide apelin as a key regulator of central and peripheral responses to homeostatic perturbations including the control of fluid homeostasis (O’Carroll and Lolait, 2003), food intake (Taheri et al., 2002) and angiogenesis (Liu et al., 2015). Apelin also has an important role in the maintenance of basic cardiac function, inducing a potent positive inotropic effect in isolated rat heart (Szokodi et al., 2002) and maintaining cardiac contractility with aging and pressure overload (Kuba et al., 2007). Additionally, apelin contributes to cardiovascular pathological processes such as heart failure (Földes et al., 2003), coronary heart disease (see Yu et al., 2014 for review) and hypertension (Lee et al., 2005; Zhang et al., 2009, 2014; Barnes et al., 2013). The specific role of the apelinergic system in neural control of cardiovascular function however, remains unclear.

Apelin (gene name *apln*), primarily synthesized as a 77-amino acid prepropeptide precursor (Tatemoto et al., 1998), is cleaved into a shorter biologically active 36 amino-acid peptide. Unlike most other G protein-coupled (GPCR) families, apelin appears to mediate its effects via binding to only one receptor subtype, the apelin receptor (APJ; gene name *aplnr*). Other isoforms of the apelin peptide are also agonists at APJ (Habata et al., 1999; Zou et al., 2000; Medhurst et al., 2003), including apelin-12, -13, and the pyroglutamyl form of apelin-13 ([Pyr^1^]apelin-13) that may be the most potent biological ligand (Tatemoto et al., 1998; Habata et al., 1999). The apelinergic system is richly expressed in central neural circuits that are involved in the modulation of BP, including the medial parvocellular paraventricular nucleus (PVN) (Lee et al., 2000; O’Carroll et al., 2000), the cerebroventricular system, and lower brainstem structures such as the rostral ventrolateral medulla (RVLM) (Lee et al., 2000; Brailoiu et al., 2002; Reaux et al., 2002). Therefore *apln* and *aplnr* transcripts are expressed in discrete brain regions that position them to be potential chronic modulators of cardiovascular tone.

While studies support apelin acting peripherally as an anti-hypertension factor (Zhong et al., 2005; Sonmez et al., 2010; Chandra et al., 2011; Zhu et al., 2013), neurological pathways profoundly influence peripheral homeostatic systems, and centrally administered [Pyr^1^]apelin-13 [the predominant apelin isoform in the cardiovascular system (Maguire et al., 2009)] has been shown to increase mean arterial blood pressure (MABP) in normotensive rats (Seyedabadi et al., 2002; Kagiyama et al., 2005; Zhang et al., 2009; Griffiths et al., 2017). Compelling evidence suggests that elevated SNA is key in the development of hypertension (Grassi, 1998; Fisher and Paton, 2012), and in the spontaneously hypertensive rat (SHR) model of hypertension, SNA is higher than in its normotensive control the Wistar-Kyoto (WKY) rat (Cabassi et al., 2002; Simms et al., 2009). Importantly, central administration of [Pyr^1^]apelin-13 is known to affect sympathetic tone in addition to MABP (Seyedabadi et al., 2002; Zhang et al., 2009; Griffiths et al., 2017), increasing SNA by mechanisms that are not understood.

The neural control of BP is mediated in part via the activity of barosensitive sympathetic efferents, set by a core network of neurons including the RVLM, the PVN and the nucleus of the solitary tract (Guyenet, 2006). A number of reports implicate apelin in the regulation of RVLM increases in SNA and MABP. In the RVLM of WKY rats, overexpression of *apln* using adeno-associated virus type 2 vector-mediated gene transfer results in a chronic elevation in BP in conscious rats (Zhang et al., 2009), and acute microinjection of apelin-13 increases BP, SNA and heart rate (HR) (Seyedabadi et al., 2002; Zhang et al., 2009) in anesthetized rats. Additionally, apelin protein levels and *apln* expression is enhanced in the RVLM of SHR compared with WKY rats, and microinjection of an apelin-neutralizing antibody into the RVLM lowers BP in anesthetized SHR but not in normotensive rats (Zhang et al., 2009), suggesting that apelin is sympathoexcitatory in this region. Moreover we have recently shown that bilateral microinjection of [Pyr^1^]apelin-13 into the RVLM increases BP and sympathetic vasomotor tone (as indirectly measured by spectral analysis of BP) in anesthetized Wistar rats (Griffiths et al., 2017).

As recent reports imply that central apelin-13 may contribute to BP regulation and play a causative role in neurogenic hypertension, we hypothesized that APJ signaling in the RVLM may contribute to the development of hypertension through regulation of SNA and that targeting this central APJ pathway may be an important new strategy in modulating sympathetic outflow to reverse or prevent hypertensive pathology. Therefore, the aim of the present study was to determine whether knockdown of APJ activity in the RVLM of hypertensive rat models acts to depress sympathetic vasomotor tone and arterial pressure. We tested this hypothesis using chronic knockdown of the RVLM APJ by lentiviral APJ-specific-small hairpin RNA (LV-APJ-shRNA) mediated gene transfer in SHR, a genetic model of essential hypertension, and *N*_ω_-Nitro-L-arginine methyl ester hydrochloride (_L_-NAME)-induced hypertensive rats, adopting radiotelemetry for long-term monitoring of multiple cardiovascular variables. We also explored whether knockdown of APJ activity in the RVLM of juvenile pre-hypertensive SHR, and thus disruption of the apelinergic system early in development, acts to prevent the onset of hypertension in this model.

## Materials and Methods

### Ethical Approval

All experiments were approved by the University of Bristol Animal Welfare and Ethical Review Body and performed in strict accordance with United Kingdom. Home Office regulations [Animals (Scientific Procedures) Act (1986)].

### Animals

Male WKY rats (WKY; *n* = 36; 225–250 g; Envigo, United Kingdom), adult SHRs(*n* = 16; 225–250 g; Animal Services Unit, University of Bristol, United Kingdom) and juvenile SHRs (*n* = 20; 90 g; Animal Services Unit, University of Bristol, United Kingdom) were housed at a constant temperature (21 ± 2°C) and humidity on a 14:10 h light:dark cycle. The light cycle was set as 0500 h lights on and 1900 h lights off. Access to standard laboratory chow and water was provided *ad libitum*. For the generation of the _L_-NAME hypertension model, where chronic oral administration of the nitric oxide synthase (NOS) inhibitor promotes persistent hypertension via overactivity of the central sympathetic nervous system (Sander and Victor, 1999; Zanzinger, 1999), blockade of NO was induced in a group of WKY rats (*n* = 6; 120 g) by oral administration of _L_-NAME (daily intake 50 mg kg^−1^ for 3 weeks; Sigma Aldrich, United Kingdom) in the drinking water to induce hypertension, followed by a maintenance dose (10 mg kg^−1^) (Guarasci and Kline, 1996; Paulis et al., 2008). Doses were calculated for each singly housed animal on the assumption that rats drink approximately 10 ml/100 g/day (Greenwood et al., 2015).

### Chronic Arterial Blood Pressure Measurement

#### Femoral Vein Cannulation

Intravenous (i.v.) cannulas were constructed by welding a 3 cm length of MRE033 (OD: 0.033,” Braintree Scientific, United Kingdom) to an 18 cm length of MRE040 tubing (OD: 0.040,” Braintree Scientific, United Kingdom). Cannula tips were coated with Tridodecylmethylammonium Chloride Heparin Complex (2% w/w; Polysciences Inc., United States). Adult WKY (*n* = 12), _L_-NAME treated WKY (*n* = 12) and SHR (*n* = 12) were anesthetized with isoflurane (4% for induction and 2% for maintenance in O_2_) and analgesia administered (Vetergesic; 0.8 mg kg^−1^). Cannulas were tunneled subcutaneously and connected at the level of the scapulas to an externalized deburred 23G needle (Coopers Needle Works, United Kingdom) that had been mounted on a 1 cm^2^ piece of polypropylene mesh (Small Parts Ltd., United States) at the level of the scapulas. On completion of surgery, rats were singly housed in a soundproofed room. Cannulas were flushed with 0.5 ml heparinized saline (50 IU ml^−1^) daily for 1 week following surgery and then every other day for the remainder of the experiment.

#### Telemeter Implantation

A radiotelemetry system (TRM54P, Millar, Houston, TX, United States) was adopted to make chronic, continuous measurements of arterial pressure as reported previously (McBryde et al., 2013). Rats underwent telemetry surgery 3 days following i.v. cannulation. Rats were anesthetized with isoflurane (as above), analgesia administered (as above), and the telemeter catheter was advanced ∼1.5 cm into the descending aorta in the peritoneal cavity above the level of the caudal aorta bifurcation. A small volume of Vetbond (WPI, United Kingdom) tissue adhesive was applied to the site of catheter entry to prevent blood leakage and the catheter was held in place using a 2 × 1 cm patch of plastic surgical mesh (Millar, New Zealand). The body of the telemeter was secured within the peritoneal cavity using non-absorbable suture material (3–0 Mersilk, Ethicon, United Kingdom). Following telemeter implantation animals were allowed 5 days recovery before blood pressure recording began. Blood pressure was recorded continuously for 5 days before (baseline) and 25 days after virus injection surgeries. Telemeters were kept charged within animals and blood pressure signals received by SmartPads (TR180; Millar). The experimental room was acoustically isolated.

### Gene Transfer Into the RVLM

#### Lentiviral Vector Generation

To produce APJ-specific knockdown, rats were transduced with a lentivirus (LV) containing short hairpin interfering RNA (shRNA) targeting the rat APJ sequence. The APJ-specific shRNA construct was generated by PCR using the pSilencer vector (10 ng), the M13 forward primer, and reverse primers designed against rat APJ (GenBank Accession NM_031349). Following amplification, the PCR product was cloned directly into the TOPO TA intermediate vector (Invitrogen, United Kingdom), transformed into TOP10 One Shot Cells (Invitrogen, United Kingdom), and clones selected and analyzed using restriction enzyme digestion with Xho1, and DNA sequencing. The APJ shRNA (LV-APJ-shRNA) and a scrambled control shRNA (LV-scr-shRNA), consisting of the same nucleotides in the shRNA but in random order and not matched to any other rat gene, were excised from pSilencer vector and expressed in the lentiviral transfer vector pRRL.SIN.CPPT.CMV.GFP.WPRE, allowing targeting of both C1 and non-C1 neurons. The lentiviral transfer vectors pRRL.SIN.CPPT.CMV.GFP. WPRE (used for determination of transduction efficiency and as a control) and pRRL.SIN. CPPT.CMV.APJ/SCR. WPRE and all packaging plasmids (pMDLg/pRRE, pRSV-Rev, PMD2.G; Addgene) were propagated in Stbl3 competent cells to reduce homologous recombination. All plasmid constructs were purified by Maxiprep using a PureLink HiPure Plasmid Filter Maxiprep kit (Invitrogen). Viruses were generated by transient transfection of the transfer vector together with 3 separate packaging plasmids into HEK293T/17 cells by the calcium phosphate method as previously described (Panyasrivanit et al., 2011). Purified virus was resuspended in prewarmed PBS (150 μl) and stored at −80°C. Viral titers were determined by counting GFP-positive cells at day 3 following infection of HEK293T cells. The effective time point after virus transfection into the RVLM was determined by a time course (16, 25, and 30 days; *n* = 9) measuring loss of [Pyr^1^]apelin-13-induced cardiovascular effects in the RVLM and was found to be maximal at 25 days.

#### Intraparenchymal Injection of Lentivirus

Ten days post telemeter implantation rats were anesthetized with an intramuscular injection of ketamine (60 mg kg^−1^) and medetomidine (250 μg kg^−1^) and analgesia administered (Vetergesic 0.8 mg kg^−1^). Rats were placed in a stereotaxic frame with the head flexed down (nose bar at −19 mm). The dorsal brainstem was exposed following partial retraction of the occipital bone and atlanto-occipital membrane. Lentivirus injections to the RVLM were made using single-barreled micropipettes (1–5 μl calibrated microcapillary tube, Sigma Aldrich, United Kingdom), angled at 20° rostral and aligned with the caudal point of the obex as the reference with the aid of a binocular surgical microscope (Leica M651, United Kingdom). The RVLM was targeted using the following stereotaxic co-ordinates: 0.18 cm lateral from midline, 0.12 cm rostral and 0.35 cm ventral for adult rats (>250 g) and 0.17 cm lateral, 0.09 cm rostral and 0.30 cm ventral for juvenile rats (∼90 g). The micropipette was advanced to the correct depth and allowed to rest for 5 min (min) prior to viral vector (1 μl, lentiviral titres: LV-APJ-shRNA, 1.12 × 10^10^ pfu ml^−1^; LV-scr-shRNA, 4.3 × 10^9^ pfu ml^−1^) injection over a period of 10 min. The pipette was then left *in situ* for a further 5 min to ensure the virus remained at the correct depth in the brainstem. This was then repeated to target the contralateral RVLM. Anesthesia was reversed by a subcutaneous injection of Antisedan (1 mg kg^−1^). Rats received injection of either LV-scr-shRNA or LV-APJ-shRNA (*n* = 6 group^−1^). Blood pressure recording continued for 25 days (see data acquisition below). The level of APJ gene knockdown was confirmed by quantitative PCR (qPCR) (see below).

### Pharmacological Manipulation of the RVLM

#### Confirmation of Chronic APJ Knockdown by Femoral Artery Blood Pressure Recording

Twenty five days post-virus injection a terminal, non-recovery, experiment was carried out to test for the long term expression of the transgene and to confirm that chronic knock-down of APJ blocked MABP responses following a direct “challenge” microinjection of [Pyr^1^]apelin-13 to the RVLM (Griffiths et al., 2017). Rats were anesthetized with sodium pentobarbital (50 mg kg^−1^ i.p.) and BP was monitored via a heparin-saline (1 U ml^−1^) filled catheter (Micro-Renathane tubing, 0.033” inner diameter, Braintree Scientific, MA, United States) implanted in the left femoral artery and connected to a pressure transducer (BD DTX Plus, Southwest Medical, Bristol, United Kingdom). Pressure signals were amplified and filtered using a NeuroLog System (Digitimer, Welwyn Garden City, United Kingdom). RVLM injections followed the same protocol described above for virus injections. The pressor response to bilateral microinjection of L-glutamate (1 nmol 100 nl^−1^ side^−1^; Tocris Bioscience, United Kingdom) was used to confirm the position of the pipette within the RVLM. Subsequent bilateral microinjections of [Pyr^1^]apelin-13 (200 pmol 100 nl^−1^; Bachem, Germany) were made at the same stereotaxic coordinates that elicited the largest L-glutamate pressor response (>20 mmHg). After the final L-glutamate microinjection, rats were euthanized using a guillotine. Brains were immediately frozen on powdered dry ice and stored at −80°C to preserve RNA integrity.

#### Acute Pharmacological Blockade of APJ in the RVLM of Anesthetized WKY and SHR

The RVLM of WKY (*n* = 4) and SHR (*n* = 4) rats was targeted bilaterally using the coordinates described previously (Griffiths et al., 2017). The position of the micropipette in the RVLM was first confirmed by a bilateral injection of L-glutamate (1 nmol 100 nl^−1^ side^−1^). Drug solutions were prepared according to the manufacturer’s instructions, diluted to the required concentration (consistent with a large body of literature on the physiological effects of drugs used in the RVLM and other brain regions (Seyedabadi et al., 2002; Zhang et al., 2009; Huber and Schreihofer, 2011; Zhang et al., 2014) in normal saline, and stock aliquots were stored at −20°C until thawed for use. Bilateral drug microinjections of 100 nl then progressed as follows; saline, [Pyr^1^]apelin-13 (200 pmol, 100 nl^−1^; Bachem, Germany), [Ala^13^]apelin-13 (F13A, 2 nmol, Phoenix Pharmaceuticals, Belmont, CA, United States), [Pyr^1^]apelin-13 (200 pmol, 100 nl^−1^), saline. A repeat injection of F13A was made ∼45 min after initial F13A injection to test any response to injection of the antagonist. Animals received a microinjection of Indian ink (100 nl; 1:10 dilution) at the end of the experiment to confirm the histological location of the micropipette within the RVLM. Animals received an anesthetic overdose and were euthanized by decapitation. Brains were removed, immediately frozen on dry ice and stored at −80°C. Hindbrains were cut at 40 μm using a cryostat (CM3050 S, Leica Microsystems) to reveal the position of ink injection relative to the RVLM.

### Tail Cuff Blood Pressure Measurement

The tail arterial systolic blood pressure was determined in juvenile SHR (90 g; *n* = 12) with a tail-cuff sphygmomanometer (CODA2 Tail Cuff Blood Pressure System; Kent Scientific, United States). Three days after stereotaxic surgery to administer LV-APJ-shRNA (*n* = 6) or LV-scr-shRNA (*n* = 6), 4-week-old juvenile SHR were acclimatized to the restraint holders, occlusion cuff, and volume pressure cuff, for between 20 and 30 min each day over the course of a week. Data acquisition was then carried out twice a week until 6 weeks after virus microinjection into the RVLM. During recording sessions rats were placed in restraint holders and the tail warmed on a warming pad for 15 min to bring the tail temperature to between 30 and 35°C (to increase blood flow and improve data acquisition). A full recording session consisted of 10 acclimatization cycles to optimize data acquisition, followed by 15 data acquisition cycles during which systolic, diastolic and mean arterial BP were monitored.

### RNA Extraction and cDNA Synthesis

Fresh, frozen sections (40 μm) were cut on a cryostat (CM3050 S, Leica Microsystems) moving in the caudal-rostral direction through the brainstem and collected on the freezing plate. Every fifth section was stained with 0.1% toluidine blue to aid in the identification of the RVLM. The RVLM is situated just caudal of the facial nucleus and as such punches were taken from the 4 sections immediately prior to the section that was first identified as having large facial nucleus cell bodies. Left and right RVLM were punched out using a 1 mm diameter micropunch (Fine Scientific Tools) and placed into separate 1.5 ml RNAse/DNAse-free tubes (Appleton Woods, United Kingdom) on dry ice and stored at −80°C, before RNA extraction. Samples were mechanically homogenized in Trizol and processed for RNA isolation and purification using the Trizol Plus RNA purification kit (Applied Biosystems, United Kingdom), according to the manufacturer’s instructions. RNA yield was measured using a Nanodrop System (Thermo Fisher Scientific, United Kingdom). For cDNA synthesis 80 ng of total RNA was treated with DNase I, to eliminate genomic DNA from the sample, prior to reverse transcription using the High Capacity cDNA Reverse Transcriptase Kit (Applied Biosystems, United Kingdom).

### Real Time qPCR Analysis

Primers for *aplnr* (5′-GCTCCCAGAGAGGCTAGTTCTG-3′ and 5′-TGGAGGCTTGGCTCAAACC-3′, bp 59–80 and 125–143, respectively, of GenBank accession NM_031349), *apln* (5′-TTCTAAAGCAGGATTGAAGGGC-3′ and 5′-CCATCAGCAGCGATAACAGG-3′, bp 569–590 and 682–701, respectively, of GenBank accession NM_031612) and the internal control housekeeping genes ribosomal protein L19 (5′-GCGTCTGCAGCCATGAGTA-3′ and 5′-TGGCATTGGCGATTTCGTTG-3′) and GAPDH (5′-ATGATTCTACCCACGGCAAG-3′ and 5′-CTGGAAGATGGTGATGGGTT-3′) were optimized and validated using standard ABI protocols. The cDNA from the reverse transcription procedure was used as template for quantitative PCR, which was carried out in duplicate in 10 μl reaction volumes using SYBR green master mix buffer (Applied Biosystems, United Kingdom). Plates (MicroAmp Fast 96-Well Reaction Plate (0.1 ml), Applied Biosystems, United Kingdom) were run for 45 cycles of 95°C for 15 s and 60°C for 1 min on a StepOnePlus real-time PCR system (Applied Biosystems, United Kingdom). All qRT-PCR reactions were followed by dissociation curve analysis. Relative quantification of gene expression was performed using the 2^ΔΔCT^ method (Livak and Schmittgen, 2001).

### Immunohistochemistry

WKY rats (*n* = 2 per group) were terminally anesthetized 25 days after virus injection using sodium pentobarbital (50 mg kg^−1^ i.p.) prior to transcardial perfusion with 4% (w/v) paraformaldehyde (PFA) in phosphate-buffered saline (PBS, pH 7.4). Brains were removed, post-fixed overnight in 4% (w/v) PFA, cryoprotected in 30% (w/v) sucrose in PBS at 4°C, flash frozen on liquid nitrogen and stored at −80°C. Sections were cut at 40 μm on a cryostat and transferred to a 24 well plate containing 1 ml PBS in each well. Every fifth section was used to confirm the expression of GFP within the RVLM. Immunohistochemical staining was performed using the free-floating method on at least 15 sections with no more than five brainstem sections per well. Sections were washed with PBS and then blocked with PBS containing 3% BSA/0.3% Triton X-100. Following blocking, sections were incubated with anti-GFP primary antibody (1:3000, rabbit polyclonal, AB290, Abcam, United Kingdom) in PBS/3% BSA/0.3% Triton X-100 at 4°C for 48 hrs, followed by secondary antibody (1:500, goat anti-rabbit AlexaFluor 488, A-11008, Thermo Fisher Scientific, United Kingdom, in 1% BSA, 0.3% Triton, PBS) at room temperature for 1 h. Sections were counterstained with DAPI (1:1000 of 1 mg/ml in PBS), mounted onto slides subbed with 0.5% (w/v) gelatin in dH_2_O, allowed to dry, and coverslipped using ProLong Gold antifade reagent (Life Tech, United Kingdom). Images were captured on an epifluorescence microscope (DMI6000 Leica Microsystems).

### Data Acquisition and Analysis

All blood pressure signals were recorded using a 1401 data acquisition system and Spike 2 version 7.14 (Cambridge Electronic Design, United Kingdom) digitized at 5 kHz. Baseline values of physiological variables, including diastolic arterial blood pressure (DABP), systolic arterial blood pressure (SABP), MABP, pulse interval (PI), respiratory rate (RR) and HR, were recorded and averaged over 5 days before the start of experiments. During chronic studies raw blood pressure waveforms were recorded for the first 10 min of each hour and BP, HR, and RR data was recorded continuously online using the HRBP version 8 script for Spike 2. Baseline data was recorded for 5 days before virus injection and for a subsequent 25 days following virus injection. BP, HR and RR data was extracted offline following acute studies. Spectral analysis of systolic BP and HR variability was performed using HRV1 script for Spike 2 to calculate power in the very low frequency (VLF): 0–0.3 Hz; LF: 0.3–0.8 Hz and high frequency (HF): 0.8–3.3 Hz frequency bands (Parati et al., 1995; Japundžić-Žigon, 1998). The following settings were used to compute the power spectra: time constant for DC removal: ± 3 s; frequency range of spectra: 0–5.12 Hz; epoch duration: 25 s; FFT size: 128; window type: Hanning. It is known that LF:HF-HRV and LF-SBPV can be used as indirect measures of cardiac and vasomotor sympathetic tone, respectively (Waki et al., 2006; Lozić et al., 2014). Additionally, to confirm that any changes in spectral analysis data between experimental groups was associated with change in sympathetic nervous activity, rats were injected with hexamethonium (i.v.; 10 mg kg^−1^) 20 days after virus injection, to directly assess the relative level of tone as indicated by the extent of the fall in arterial pressure. The change in blood pressure following hexamethonium injection was calculated by subtracting MABP over the 10 s period following injection from a 10 s baseline period. BP and pulse interval waveforms were also analyzed to assess changes in baroreflex sensitivity (BRS; Oosting et al., 1997) using sBRG script (Spike 2, CED). BRS values calculated over a total 24 h recording period were averaged to provide a single value per day. All data are presented as mean ± SEM unless otherwise stated. Statistical analysis was carried out using Graphpad Prism 5.0 software for Mac (Graphpad Software Inc., United States). Statistical significance for differences between groups (*n* = 6) was defined as *P* < 0.05. Responses following injection of pharmacological compounds to the RVLM were compared using a 1-way analysis of variance (ANOVA) with Tukey post-tests. Time course data was compared using a 2-way analysis of variance (ANOVA) with Bonferroni Post-tests. Otherwise statistical differences between two experimental groups were evaluated using independent-sample unpaired Student’s *t*-tests.

## Results

### Upregulation of *apln* and *aplnr* Expression in RVLM of SHR

**FIGURE 1:**
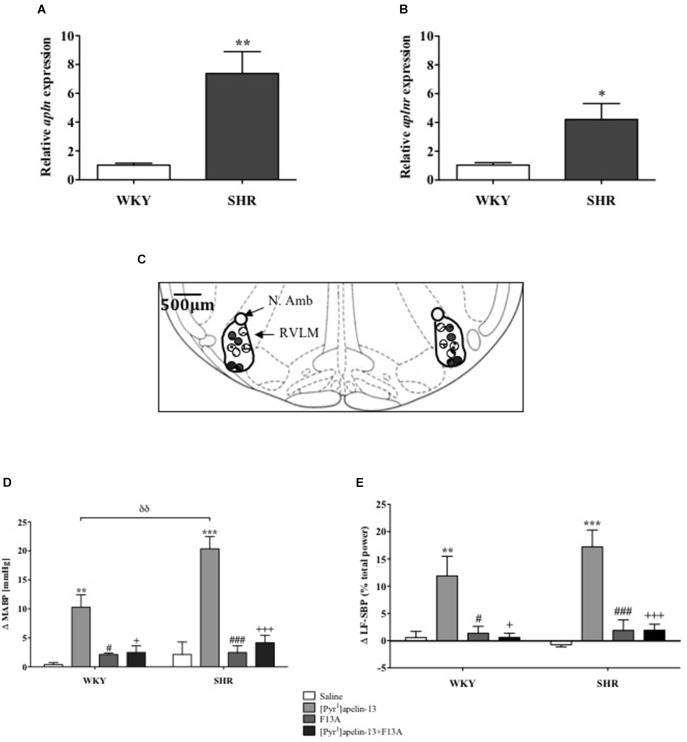
The apelinergic system is upregulated in SHR. Relative expression of **(A)** *apln* and **(B)** *aplnr* in RVLM micropunches from WKY (*n* = 4) and SHR (*n* = 4) rats quantified by RT-PCR. Data shown is mean ± SEM. ^∗^*P <* 0.05, ^∗∗^*P <* 0.01. **(C)** Schematic illustrating the localisation of injection sites (matching dots indicate bilateral injection sites), representative of sections from each animal group, determined by examination of the deposition of dye in the brain stem of animals. RVLM and N. Ambiguus (N. Amb) are indicated (Adapted from Paxinos and Watson, 1998; Figure 68). **(D,E)**, change in **(D)** MABP and **(E)** LF-SBP following microinjection of saline, [Pyr^1^]apelin-13, F13A and F13A + [Pyr^1^]apelin-13 to the RVLM of WKY (*n* = 4) and SHR (*n* = 4) rats. Data shown is mean ± SEM. ^∗∗^*P <* 0.01, ^∗∗∗^*P <* 0.001, saline vs. [Pyr^1^]apelin-13; ^#^*P <* 0.05, ^###^*P <* 0.001, F13A vs. [Pyr^1^]apelin-13; + *P <* 0.05, + + + *P <* 0.001, [Pyr^1^]apelin-13 vs. F13A + [Pyr^1^]apelin-13; ^δδ^ *P <* 0.01, WKY [Pyr^1^]apelin-13 vs. SHR [Pyr^1^]apelin-13.

*Apln* and *aplnr* expression levels in micropunches of RVLM from SHR and WKY rats were quantified using qPCR (*n* = 4/group). Both *apln* (*P <* 0.01) and *aplnr* (*P <* 0.05) transcript levels were significantly higher in SHR in comparison with WKY control rats (Figures 1A,B, respectively).

### Effects of Acute APJ Blockade on Cardiovascular Parameters in Anesthetized Normotensive WKY and Hypertensive SHR Rats

Baseline MABP was significantly increased in SHR compared to WKY rats (130 ± 14 mmHg vs. 87 ± 3 mmHg, respectively, *P <* 0.05, *n* = 4/group). Figure 1C shows a schematic illustrating the localization of injection sites. Microinjection of [Pyr^1^]apelin-13 (200 pmol) to the RVLM increased MABP and LF-SBP in the RVLM of WKY (10 ± 2 mmHg, *P* < 0.01; 11.9 ± 3.6%, *P* < 0.05, respectively) and SHR (20 ± 2 mmHg, *P* < 0.01, 17.2 ± 3.1%, *P* < 0.05, respectively) compared to saline-injected controls (WKY:0.4 ± 0.3 mmHg and 0.6 ± 1.1%; SHR: 2.1 ± 2.2 mmHg and −0.7 ±−0.4%; Figures 1D,E, respectively). The pressor response to microinjection of [Pyr^1^]apelin-13 in the RVLM of SHRs was significantly greater than in WKY (*P* < 0.01, Figure 1D). Microinjection of an APJ-specific antagonist, [Ala^13^]-apelin-13 (F13A, 2 nmol), alone into the RVLM had no significant effect on BP or LF-SBP in either WKY or SHR, but abolished all [Pyr^1^]apelin-13-mediated increases in BP and LF-SBP in WKY and SHR (MABP: 2 ± 1 mmHg and 4 ± 1 mmHg, respectively, Figure 1D; LF-SBP: 0.6 ± 0.8% and 1.9 ± 1.1% respectively, Figure 1E). Microinjection of [Pyr^1^]apelin-13 into the RVLM of WKY and SHR had no statistically significant effect on HR.

### Effects of LV-APJ-shRNA Microinjection on *aplnr* Expression and Function

**FIGURE 2:**
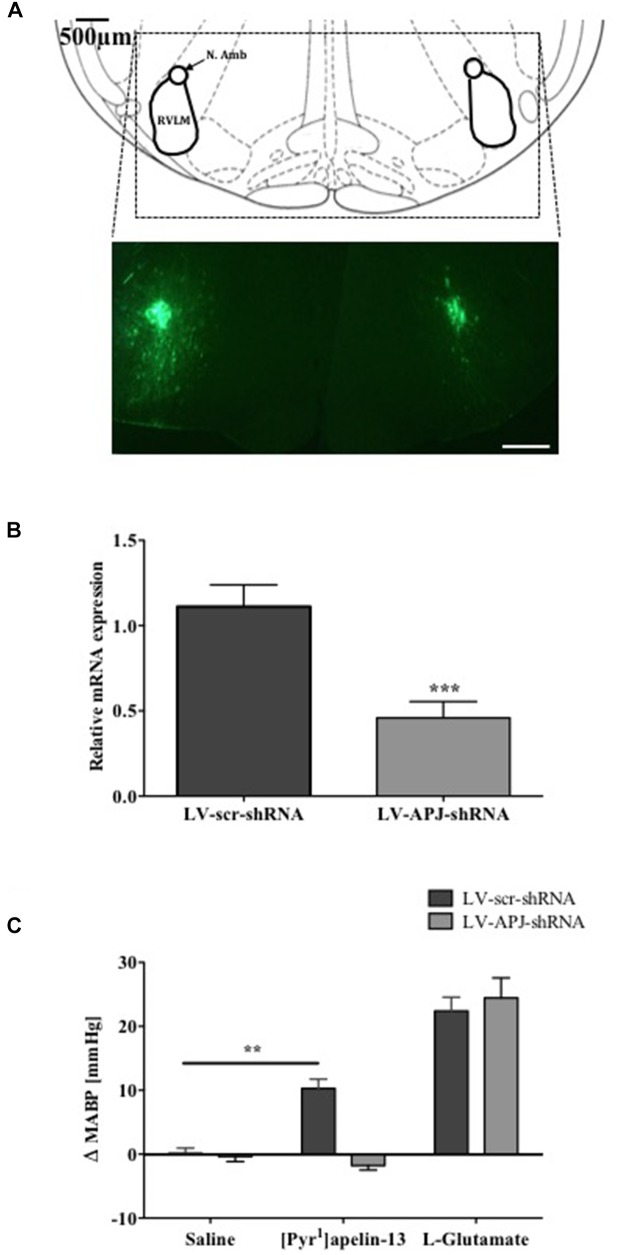
Effects of LV-APJ-shRNA microinjection on *aplnr* expression and function. **(A)** Schematic diagram showing the anatomical location of the RVLM in the brainstem targeted with lentivirus injections, adapted from Paxinos and Watson (1998, Figure 68) and representative fluorescent, photomicrographs showing eGFP expression within the RVLM of a WKY rat 25 days after virus injection. Scale bar = 500 μm. **(B)** Relative *aplnr* expression 25 days after bilateral microinjection of either LV-scr-shRNA (dark gray bar, *n* = 18) or LV-APJ-shRNA (light gray bar, *n* = 18) to the RVLM. **(C)** Change in MABP following bilateral microinjection of saline, [Pyr^1^]apelin-13 (200 pmol, 100 nl^−1^ side^−1^) and glutamate (1 nmol, 100 nl^−1^) to the RVLM of LV-scr-shRNA, (dark gray bars, *n* = 18) and LV-APJ-shRNA injected (light gray bars, *n* = 18) rats. Data shown is mean ± SEM ^∗∗^*P <* 0.01, ^∗∗∗^*P <* 0.001.

To investigate the role of APJ in the RVLM for BP control, lentiviral vector-mediated gene delivery was used to knockdown *aplnr* in the RVLM. Immunofluorescence confocal microscopy confirmed expression of eGFP fluorescence, and thus localization of eGFP protein and the transgene, in the RVLM within 300 μm of the injection sites of virus-injected male WKY rats at 25 days post-virus injection (Figure 2A), supporting long-term expression of the transgene. qPCR analysis showed successful knockdown of *aplnr* in the RVLM of rats injected with LV-APJ-shRNA when compared with LV-scr-shRNA-injected rats (Figure 2B; *P* < 0.05). LV-APJ-shRNA decreased *aplnr* expression by ∼64% at day 25 post-virus injection in punches from the RVLM of LV-APJ-shRNA-injected rats. Additionally, to confirm APJ knockdown efficiency *in vivo*, the cardiovascular responses of rats to a direct “challenge” microinjection of [Pyr^1^]apelin-13 to the RVLM were measured 25 days after virus transduction. As expected, bilateral microinjection of [Pyr^1^]apelin-13 into the RVLM of LV-scr-shRNA-injected rats elicited an increase in mean basal BP of 10.3 ± 1.4 mmHg (Figure 2C), and these increases were completely abrogated in LV-APJ-shRNA-injected rats (−1.7 ± 1.4 mmHg, *P* < 0.01). However, microinjection of L-glutamate, used to assess functionally the position of the micropipette in the RVLM at the beginning of each experiment (*n* = 12), caused a significant and comparable increase in MABP in LV-scr-shRNA- and LV-APJ-shRNA-injected rats (22.4 ± 2.1 mmHg and 24.5 ± 3.1 mmHg, respectively; Figure 2C) when compared to saline controls (0.1 ± 0.8 mmHg and −0.4 ± 0.8 mmHg, respectively, *P* < 0.001; Figure 2C).

### Effects of Chronic *aplnr* Knockdown in the RVLM on Cardiovascular Parameters in WKY Rats

**FIGURE 3:**
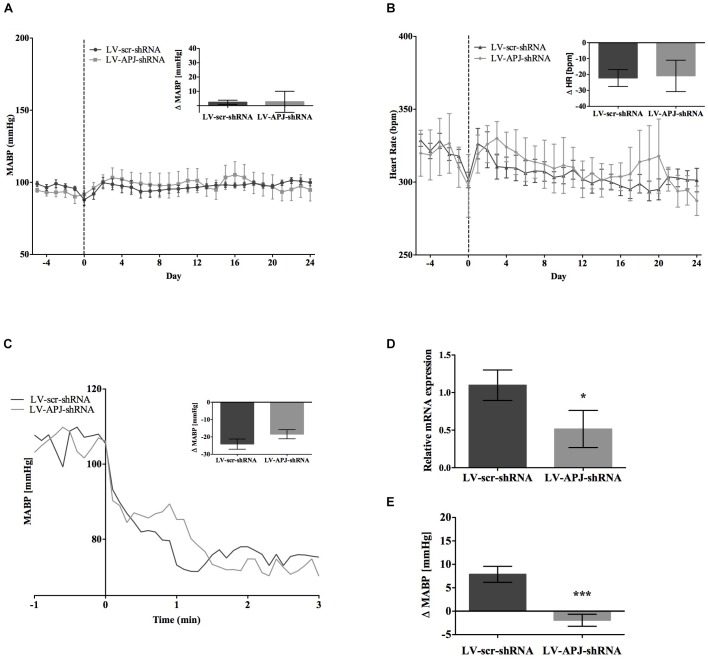
Effects of knockdown of *aplnr* on cardiovascular parameters in WKY rats. Changes in **(A)** MABP and **(B)** HR ranging from 5 days before (–5) to 25 days after bilateral microinjection (vertical dotted line) of either LV-scr-shRNA (*n* = 6) or LV-APJ-shRNA (*n* = 6). Inset bar graphs show change in **(A)** MABP or **(B)** HR and calculated as MABP or HR at day –5 subtracted from MABP or HR at day 24. **(C)** Blockade of autonomic ganglionic transmission (hexamethonium 10 mg kg^−1^, i.v.) produced no difference in MABP response in LV-APJ-shRNA (*n* = 6) or LV-APJ-shRNA (*n* = 6) injected WKY. **(D)** Relative *aplnr* expression in the RVLM and **(E)** change in MABP following bilateral administration of [Pyr^1^]apelin-13 (200 pmol) to the RVLM of LV-scr-shRNA (dark gray bar, *n* = 6) or LV-APJ-shRNA (light gray bar, *n* = 6), 25 days after virus injection. Data shown is mean ± SEM. ^∗^*P <* 0.05, ^∗∗∗^*P <* 0.001.

Baseline physiological variables in awake normotensive WKY rats are summarized in Table 1. No significant difference was seen in MABP or HR between LV-APJ-shRNA- and LV-scr-shRNA-transduced WKY groups at any time point over the 25-day monitoring period (Figures 3A,B respectively). Hexamethonium-induced falls in arterial pressure were similar between the LV-APJ-shRNA- and the LV-scr-shRNA-transduced WKY groups (Figure 3C). Body weights of LV-APJ-shRNA- and LV-scr-shRNA-transduced WKY groups increased comparably (46 ± 3g and 50 ± 2 g, respectively) over the 25-day monitoring period. The effectiveness of LV-APJ-shRNA-injection was verified with a challenge microinjection of [Pyr^1^]apelin-13 into the RVLM of anesthetized WKY rats at day 25 post-virus injection, where knockdown of *apnlr* prevented the increase in MABP in response to microinjection of [Pyr^1^]apelin-13 (Figure 3D *P* < 0.001). Additionally qPCR analysis of *aplnr* showed knockdown of *aplnr* in the RVLM of rats injected with LV-APJ-shRNA when compared with LV-scr-shRNA-injected rats (Figure 3E; *P* < 0.05). Similarly LF-SBP, LF:HF HR and BRG were not significantly different between LV-APJ-shRNA- and LV-scr-shRNA-transduced WKY groups at any time point (Table 2).

**Table 1:** Baseline physiological parameters measured prior to virus injection in WKY, SHR, and _L_-NAME treated WKY rats.

	WKY		SHR		_L_-NAME-treated	
	LV-scr-shRNA	LV-APJ shRNA	LV-scr-shRNA	LV-APJ-shRNA	LV-scr-shRNA	LV-APJ-shRNA
DABP (mmHg)	79 ± 2	76 ± 4	101 ± 6	98 ± 5	101 ± 13	102 ± 12
SABP (mmHg)	121 ± 2	116 ± 3	160 ± 6	167 ± 11	155 ± 13	160 ± 15
MABP (mmHg)	98 ± 2	93 ± 3	130 ± 2	127 ± 5	126 ± 13	129 ± 13
PP (mmHg)	42 ± 1	40 ± 2	60 ± 1	60 ± 1	54 ± 1	57 ± 4
RR (breaths/min)	94 ± 2	86 ± 1	87 ± 2	87 ± 2	94 ± 2	93 ± 3
HR (bpm)	323 ± 4	320 ± 17	337 ± 15	324 ± 8	325 ± 14	305 ± 2
Weight (g)	261 ± 4	273 ± 9	289 ± 4	292 ± 9	233 ± 6	233 ± 5

**Table 2:** Knockdown of *aplnr* in the RVLM of WKY, SHR, and _L_-NAME treated WKY rats had no effect on LF-SBP (% total power), LF:HF HR or baroreflex sensitivity (BRS, ms/mmHg).

	LV-scr-shRNA			LV-APJ-shRNA		
	LF-SBP (% of total power)					
	Baseline	Day 24	Change	Baseline	Day 24	Change
WKY	9.43 ± 0.72	6.63 ± 0.52	−3.00 ± 1.01	9.04 ± 1.62	6.90 ± 0.70	−1.92 ± 1.25
SHR	13.68 ± 0.76	10.19 ± 0.47	−3.84 ± 0.65	12.32 ± 1.04	9.77 ± 0.69	−2.84 ± 1.55
_L_-NAME	8.92 ± 0.23	5.50 ± 0.26	−3.63 ± 0.29	7.34 ± 0.61	4.74 ± 0.26	−2.70 ± 0.45

	**LF:HF HR**					
	**Baseline**	**Day 24**	**Change**	**Baseline**	**Day 24**	**Change**

WKY	0.29 ± 0.08	0.27 ± 0.05	−0.02 ± 0.03	0.22 ± 0.03	0.25 ± 0.07	0.02 ± 0.05
SHR	0.22 ± 0.02	0.19 ± 0.01	−0.04 ± 0.02	0.22 ± 0.05	0.26 ± 0.09	0.04 ± 0.09
_L_-NAME	0.20 ± 0.01	0.21 ± 0.04	0.02 ± 0.04	0.24 ± 0.07	0.20 ± 0.01	−0.05 ± 0.07

	**BRS (ms/mmHg)**					
	**Baseline**	**Day 24**	**Change**	**Baseline**	**Day 24**	**Change**

WKY	0.60 ± 0.09	0.57 ± 0.05	0.01 ± 0.09	0.67 ± 0.03	0.63 ± 0.10	−0.02 ± 0.09
SHR	0.36 ± 0.03	0.31 ± 0.04	0.02 ± 0.02	0.42 ± 0.03	0.44 ± 0.11	0.06 ± 0.09
_L_-NAME	0.53 ± 0.10	0.51 ± 0.07	−0.01 ± 0.09	0.52 ± 0.07	0.37 ± 0.04	−0.14 ± 0.03

### Effects of Chronic *aplnr* Knockdown in the RVLM on Cardiovascular Parameters in SHR and _L_-NAME-Treated Rats

**FIGURE 4:**
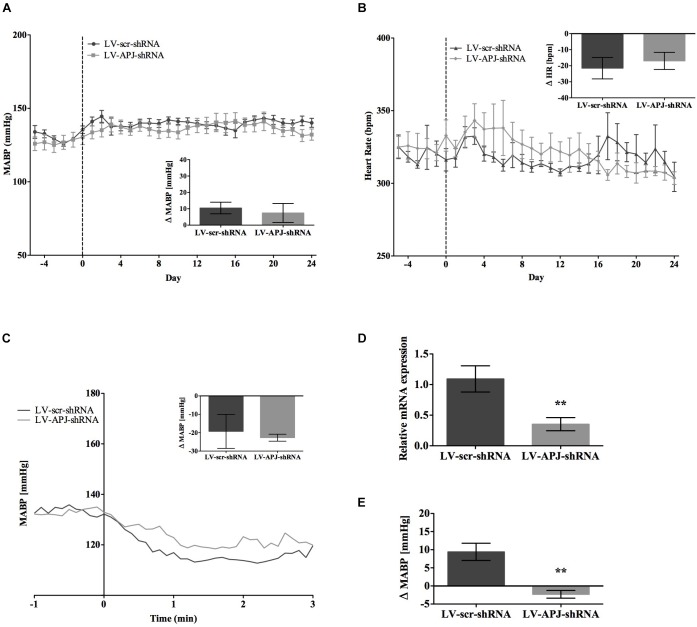
Effects of knockdown of *aplnr* on cardiovascular parameters in SHRs. Changes in **(A)** MABP and **(B)** HR ranging from 5 days before (–5) and 25 days after bilateral microinjection (vertical dotted line) of either LV-scr-shRNA (*n* = 6) or LV-APJ-shRNA (*n* = 6). Inset bar graphs show change in **(A)** MABP or **(B)** HR calculated as MABP or HR at day –5 subtracted from MABP or HR at day 24. **(C)** Blockade of autonomic ganglionic transmission (hexamethonium 10 mg kg^−1^, i.v.) produced a no difference in MABP response in LV-scr-shRNA (*n* = 6) or LV-APJ-shRNA (*n* = 6) injected SHR. **(D)** Relative *aplnr* expression in the RVLM and **(E)** change in MABP following bilateral administration of [Pyr^1^]apelin-13 (200 pmol) to the RVLM of LV-scr-shRNA (dark gray bar, *n* = 6) or LV-APJ-shRNA (light gray bar, *n* = 6), 25 days after virus injection. Data shown is mean ± SEM. ^∗∗^*P <* 0.01.

**FIGURE 5:**
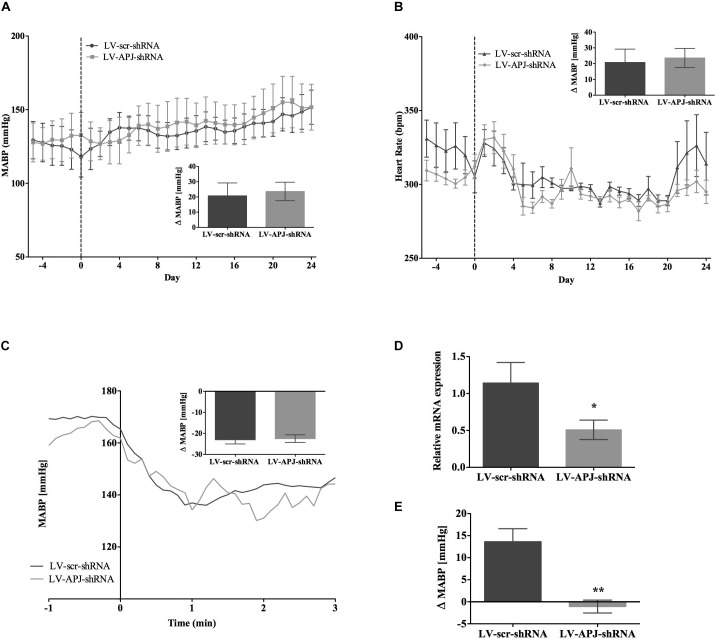
Effects of knockdown of *aplnr* on cardiovascular parameters in _L_-NAME treated WKY rats. Changes in **(A)** MABP and **(B)** HR ranging from 5 days before (–5) and 25 days after bilateral microinjection (vertical dotted line) of either LV-scr-shRNA (*n* = 6) or LV-APJ-shRNA (*n* = 6). Inset bar graphs show change in **(A)** MABP or **(B)** HR calculated as MABP or HR at day –5 subtracted from MABP or HR at day 24. **(C)** Blockade of autonomic ganglionic transmission (hexamethonium 10 mg kg^−1^, i.v.) produced a no difference in MABP response in LV-scr-shRNA (*n* = 6) or LV-APJ-shRNA (*n* = 6) injected SHR. **(D)** Relative *aplnr* expression in the RVLM and **(E)** change in MABP following bilateral administration of [Pyr^1^]apelin-13 (200 pmol) to the RVLM of LV-scr-shRNA (dark gray bar, *n* = 6) or LV-APJ-shRNA (light gray bar, *n* = 6), 25 days after virus injection. Data shown is mean ± SEM. ^∗^*P <* 0.05, ^∗∗^*P <* 0.01.

SHR and _L_-NAME-treated rats mice exhibited higher SABP, DABP, MABP, and PP at the beginning of experiments compared with normotensive WKY rats, while RR and HR were similar between normotensive and hypertensive animals (Table 1). In LV-APJ-shRNA rats MAPB and HR was unchanged compared to rats receiving LV-scr-shRNA; this was the case in both SHR (Figures 4A,B) and _L_-NAME-treated rats (Figures 5A,B). No change was seen in hexamethonium-induced falls in arterial pressure between the LV-APJ-shRNA- and the LV-scr-shRNA-transfected SHR or _L_-NAME groups (Figures 4C, 5C respectively). No significant difference was seen in body weights between the LV-APJ-shRNA- and LV-scr-shRNA-transduced SHR (LV-APJ-shRNA: 32 ± 3g; LV-scr-shRNA: 32 ± 4g, *P* > 0.05) or _L_-NAME (LV-APJ-shRNA: 40 ± 10g; LV-scr-shRNA: 47 ± 3g, *P* > 0.05) groups over the 25-day monitoring period.

A subsequent challenge microinjection of [Pyr^1^]apelin-13 into the RVLM at day 25 post LV-APJ-shRNA injection resulted in a significantly reduced response in MABP in both anesthetized SHR and _L_-NAME-treated rats (Figures 4D, 5D). Additionally qPCR analysis of *aplnr* showed knockdown of *aplnr* in the RVLM of rats injected with LV-APJ-shRNA when compared with LV-scr-shRNA-injected rats (Figures 4, 5E; *P* < 0.01 and *P* < 0.05, respectively). Similarly no significant differences were seen in LS-SBP, LF:HF HR and BRG between LV-APJ-shRNA- and LV-scr-shRNA-transduced SHR and _L_-NAME-treated groups (Table 2).

### APJ in the Development of Hypertension in Pre-hypertensive Juvenile SHR

**FIGURE 6:**
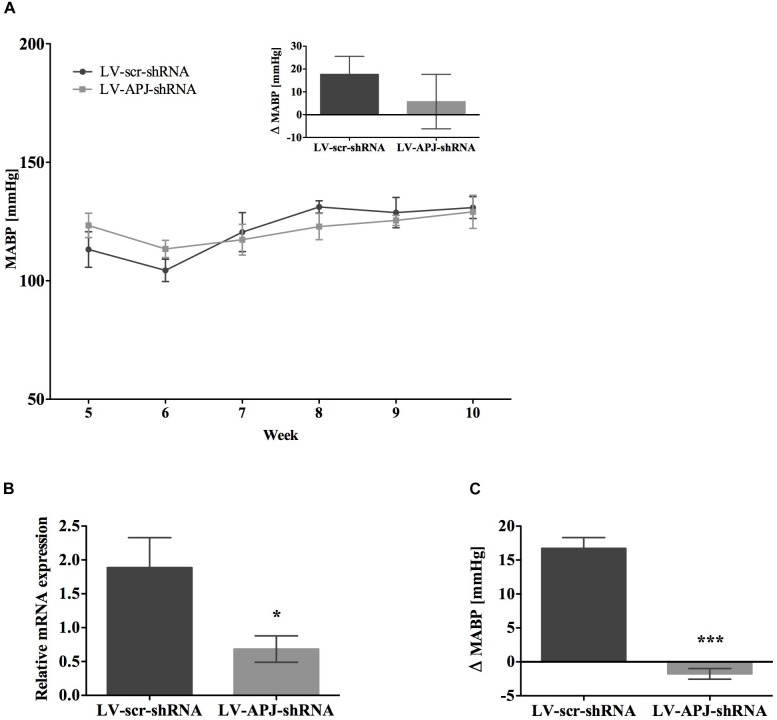
Effects of knockdown of *aplnr* in pre-hypertensive SHR. **(A)** Changes in MABP in juvenile SHR after bilateral microinjection of either LV-scr-shRNA (*n* = 6) or LV-APJ-shRNA (*n* = 6) at 4 weeks. Inset bar graph shows change in MABP calculated as MABP at 5 weeks subtracted from MABP at 10 weeks. **(B)** Relative *aplnr* expression in the RVLM and **(C)** change in MABP following bilateral administration of [Pyr^1^]apelin-13 (200 pmol) to the RVLM of LV-scr-shRNA (dark gray bar, *n* = 6) or LV-APJ-shRNA (light gray bar, *n* = 6) 6 weeks after virus injection. Data shown is mean ± SEM. ^∗^*P <* 0.05, ^∗∗∗^*P <* 0.001.

To attenuate the expression of *aplnr* in pre-hypertensive SHR, LV-APJ-shRNA was microinjected into juvenile 4-week-old SHRs immediately prior to the crucial period between 4 and 8 weeks of age when hypertension develops (Dickhout and Lee, 1998). As evidenced using tail-cuff BP measurements, the progression of hypertension in SHRs treated with LV-APJ-shRNA was similar to animals microinjected with control LV-scr-shRNA (Figure 6A), showing increased levels of BP characteristic of untreated SHR. *Aplnr* knockdown by LV-APJ-shRNA transduction was confirmed, at the end of the experiment, by the decreased expression of *aplnr* in the RVLM (Figure 6B) and the lack of response to microinjection of [Pyr^1^]apelin-13 in the RVLM when compared to the increase in MABP observed in response to microinjection of [Pyr^1^]apelin-13 in LV-scr-shRNA-transduced rats (Figure 6C).

## Discussion

Chronic sympathetic hyperactivity is present in a significant percentage of patients with essential hypertension, responsible for 95% of all hypertensive disorders (Carretero and Oparil, 2000), but the causative mechanisms remain unclear. The RVLM is a major brain region involved in the regulation of SNA associated with cardiovascular function, and recent work has shown that bilateral microinjection of [Pyr^1^]apelin-13 into this region increases sympathetic vasomotor tone and BP (Zhang et al., 2009). We hypothesized therefore, that APJ signaling in the RVLM may contribute to the development of hypertension. Our study however, examining the physiological outcome of chronic knockdown of *aplnr* in the RVLM on cardiovascular regulation in conscious, unrestrained, WKY, SHR, or _L_-NAME-treated WKY rats, reveals that MABP and SNA is similar with or without the integrity of *aplnr*. These results indicate that chronic decreased *aplnr* expression in the RVLM does not influence endogenous apelin-mediated MABP or sympathetic vasomotor drive in these normotensive and hypertensive rodents. Additionally we show that knockdown of *aplnr* in the RVLM of pre-hypertensive SHR does not prevent or delay the age-dependent development of hypertension in the SHR. Thus, apelin activation of APJ does not appear to have a role to play in the genesis or maintenance of hypertension in the animal models used in this study.

A recent study has suggested that apelin levels are enhanced in the RVLM of hypertensive SHR, and additionally, that overexpression of *apln* in the RVLM of WKY rats by gene transfer chronically increases MABP (Zhang et al., 2009). However, although apelin has been thought to contribute to the establishment of hypertension (Seyedabadi et al., 2002; Chandra et al., 2011; Zhu et al., 2013), the importance of the apelinergic system in influencing SNA and arterial BP is unclear. Recent work by Zhang et al. (2014) indicates that both apelin and APJ gene and protein expression are up-regulated in the PVN in SHR, and that pharmacological inhibition of APJ following microinjection of the APJ-specific antagonist F13A in the PVN decreases SNA and BP in SHR. These observations suggest that APJ in the PVN contributes to hypertension via modulation of sympathetic activation. In the present study we confirm the finding of Zhang et al. (2009) showing an enhanced expression of *apln* in the RVLM of SHR, and furthermore we show a significant upregulation of *aplnr* in the RVLM of this model of hypertension, in comparison to WKY rats. Additionally we show a greater acute MABP response to the microinjection of [Pyr^1^]apelin-13 in the RVLM of SHR than that seen in WKY rats, reinforcing the premise that an augmented expression of the apelinergic system in the RVLM of hypertensive animals may have a role to play in influencing MABP and sympathetic vasomotor tone.

The possible role of APJ in cardiovascular regulation has been investigated primarily in acute (<30 min) experimental conditions in anesthetized animals (Seyedabadi et al., 2002; Mitra et al., 2006; Zhang et al., 2009), and in the present study we have corroborated these studies and have shown that acute activation of APJ by exogenous administration of [Pyr^1^]apelin-13 into the RVLM increases MABP and SNA, as measured by spectral analysis, in SHR and WKY rats. Administration of the APJ antagonist, F13A, into the RVLM prevented [Pyr^1^]apelin-13-induced increases in MABP in both SHR and WKY rats, thus suggesting a role for APJ in MABP regulation. However, administration of F13A alone had no significant effect on MABP in SHR, showing that, despite the upregulation of *apln* and *aplnr* in the RVLM of SHR, there is no tonic (i.e., endogenous) activation of APJ in the RVLM contributing to MABP in these animals. F13A administration alone in the RVLM also had no significant effect on MABP and SNA in WKY rats. This is consistent with our previous acute study in the RVLM of normotensive Wistar rats (Griffiths et al., 2017) but is inconsistent with previous studies where injection of an apelin-neutralizing antibody into the RVLM significantly decreased BP in SHR (Zhang et al., 2009), or with a study in the PVN where APJ was shown to contribute to both tonic sympathetic activity and to SNA and BP increases induced by PVN-apelin-13-microinjection in SHR (Zhang et al., 2014).

Following on from our acute studies, our purpose was to chronically knockdown *aplnr* expression in RVLM neurons and to evaluate the resultant consequences upon long-term SNA and BP regulation in conscious normotensive and hypertensive rats. To that end, for the first time to our knowledge, a LV-APJ-specific shRNA construct (LV-APJ-shRNA) was generated that was shown to successfully obliterate the APJ RVLM pressor response to apelin *in vivo* after microinjection into the RVLM. Immunofluorescence confocal microscopy confirmed expression of the LV-APJ-shRNA transgene in the RVLM in microinjected rats. Additionally, in all experimental groups, the injection of LV-APJ-shRNA to the RVLM successfully blocked the MABP and SNA effects of exogenously applied [Pyr^1^]apelin-13, confirming long-term *aplnr* knockdown in the RVLM that results in a high efficacy of silencing of [Pyr^1^]apelin-13-mediated APJ pressor effects, and indicating that the cardiovascular effects of exogenous apelin are mediated specifically via activation of APJ. Thus we are confident that APJ in the RVLM has been successfully targeted in our studies.

Our results show that chronic decreased *aplnr* expression in the RVLM of WKY, SHR, or _L_-NAME-treated WKY rats after LV-APJ-shRNA microinjection did not result in sympathoinhibition, or in a significant decrease in MABP, or in HR, when compared to control LV-scr-shRNA-injected rats. It must be noted that our studies of SNA changes are indirect and based on spectral analysis. However, injection of hexamethonium produced a similar fall in arterial pressure in LV-APJ-shRNA-transfected animals compared with LV-scr-shRNA-transfected animals across all experimental groups, suggesting that the basal level of sympathetic vasomotor tone was unchanged by knockdown of *aplnr* expression and APJ activity. We do not know the extent to which changes in *aplnr* expression are mirrored at the protein level - however, if residual APJ protein is present following chronic *aplnr* knockdown it is not sufficient to elicit a cardiovascular response to exogenously applied apelin. Our results indicate that, in agreement with our acute studies, endogenous APJ activity in the RVLM is not involved in the long-term tonic control of SNA and BP in conscious WKY, SHR or _L_-NAME-treated WKY rats. We also investigated whether attenuation of the expression of *aplnr*, and thus levels of functional APJ protein, in the RVLM of pre-hypertensive SHR disrupts the development of hypertension. BP has been shown to begin to diverge between SHR and WKY rats at 4 weeks of age (Dickhout and Lee, 1998). However, the development of hypertension in juvenile SHR treated with LV-APJ-shRNA at 4 weeks of age, as evidenced using tail-cuff BP measurements, was comparable to LV-scr-shRNA-injected rats and was characteristic of levels seen in control SHR. These results indicate that chronic *aplnr* knockdown conducted at a pre-hypertensive age had no role in the onset or eventual degree of hypertension in SHR, and did not delay age-dependent progression of hypertension.

As the lentivirus microinjection in our study clearly targets the RVLM and blocks APJ functional activity, possible explanations for the discrepancies between our results and those of other studies may be the conscious state of the animals, different effects of the apelin isoforms used, or may be a reflection of the regions studied (PVN vs. RVLM). Acute studies used anesthetized rats whereas in contrast our chronic study was conducted in conscious, unrestrained rats. It is well documented that anesthesia modifies cardiovascular responses mediated via SNA (Lake et al., 1997), and conflicting results about the role of peripheral apelin in regulating vascular tone have been suggested to depend on the presence or absence of anesthesia - in anesthetized intact rats peripherally injected apelin has a hypotensive action (Lee et al., 2000; Reaux et al., 2001; Mitra et al., 2006), but has no consistent effect in awake, unrestrained rats (Kagiyama et al., 2005; Mitra et al., 2006). However, our chronic studies in conscious animals are in agreement with our acute results in anesthetized rats, indicating no contribution of endogenous apelin in the RVLM to hypertension via activation of SNA and MABP. It is of note that physiological findings (e.g., on BP control, cardiac homeostasis) in *apln* and *aplnr* KO mice have suggested that another endogenous APJ ligand, or additional apelin receptors may exist (Kuba et al., 2007; Charo et al., 2009). Therefore we cannot discount (1) that some unidentified endogenous ligand may be acting at residual APJ, remaining in the RVLM after LV-APJ-shRNA microinjection, to elicit a cardiovascular response or (2) that apelin may act at a receptor other than APJ in the RVLM to contribute to elevated SNA and BP in WKY, SHR, or _L_-NAME-treated WKY rats. It remains possible that the pressor effect of apelin may originate, in part, from its effects on the PVN or from other brain regions.

Apelin/APJ is upregulated in a number of (patho)physiological conditions including chronic heart failure (Földes et al., 2003), hypoxia (Geiger et al., 2011), insulin and obesity (Boucher et al., 2005; Geurts et al., 2011), exercise (Zhang et al., 2006) and stress (O’Carroll et al., 2003). However, despite the fact that *aplnr* (and *apln*) gene expression is upregulated in the RVLM of SHR, it does not appear to be coupled to the tonic control of MABP. Increased *aplnr/apln* expression in the RVLM does not automatically reflect increased APJ and apelin protein levels in this region - the increase in RVLM transcripts may result in increased APJ and apelin at sites distal to the RVLM, on projecting axon terminals, and not within the RVLM itself. It will be critical in future studies to determine whether the increase in RVLM *aplnr*/*apln* is associated with an increase in APJ/apelin protein. Additionally, it should be noted that an increase in receptor density in itself does not necessarily equate to a change in receptor sensitivity and/or functional responses (Larsson et al., 1989). It is also possible that this transcript upregulation is an indirect result of changes in other transmitter systems, that project to or are inherent to the RVLM, and that these changes override any contribution of the apelinergic system to the maintenance of MABP. BP regulation is a complex physiological function dependent on the interaction between a broad range of chemical mediators including local endothelial-derived factors and the cardiovascular, neural and renal-endocrine systems (Chopra et al., 2011). Previous studies in other receptor systems have shown compensatory changes in gene expression following injection of gene-specific lenti- or adenoviral constructs (Chen et al., 2006; Blume et al., 2013). APJ interacts with other receptors to regulate the cardiovascular system (Gurzu et al., 2006; Rostamzadeh et al., 2017, 2018), and forms heterodimers *in vitro* with other GPCRs e.g., the angiotensin II receptor (Chun et al., 2008), the kappa opioid receptor (Li et al., 2012), the neurotensin 1 receptor (Bai et al., 2014a) and the bradykinin 1 receptor (Bai et al., 2014b), to selectively regulate cellular responses mediated by apelin. It is tempting to speculate that the V1a vasopressin receptor (V1aR), a key modulator of cardiovascular regulation (Japundžić-Žigon, 2013), might be capable of forming heterodimers with APJ. Apelin and APJ have been described as counter-regulators of the vasopressinergic system and, in the hypothalamo-neurohypophysial system, the physiological effects of apelin appear to be mediated through modulation of vasopressin neuron activity and secretion (Reaux et al., 2001; Taheri et al., 2002; De Mota et al., 2004; Reaux-Le Goazigo et al., 2004; Tobin et al., 2008). Both APJ and V1aRs are expressed in the RVLM (Kc et al., 2010) and moreover, we have shown that [Pyr^1^]apelin-13 acts as a modulating neurotransmitter in the normotensive RVLM, interacting with the V1aR to affect vascular tone, thus indicating an interplay between the apelinergic and vasopressinergic systems at the level of the RVLM (Griffiths et al., 2017). Conceivably APJ and V1aR may interact to regulate cardiovascular function, and a loss of APJ activity within the RVLM may influence the expression or cellular signaling of V1aR to cause homeostatic adaptation. However, such a direct functional relationship of APJ with V1aR, or any other receptor system, requires further evaluation.

In summary, our data establish that *aplnr* in the RVLM does not play a role in the maintenance or development of chronic elevated BP in the SHR genetic model of high BP, or in _L_-NAME-treated WKY rats. A long-term BP regulatory function for APJ may become apparent only in other pathophysiological conditions and it remains to be established whether APJ may play a critical role in other models of hypertension such as deoxycorticosterone acetate-salt, that activates both the vasopressinergic and sympathetic nervous systems, or angiotensin II-induced hypertensive rats.

## Author Contributions

A-MO’C, SL, and JP were responsible for acquisition of funding. A-MO’C, FM, SL, and JP contributed to the conception and design of the research. PG and LP performed experiments. A-MO’C, SL, and PG interpreted results of experiments. A-MO’C and PG drafted the manuscript. A-MO’C, JP, SL, FM, LP, and PG edited and revised the manuscript. All authors have approved the final version of the manuscript and agreed to be accountable for all aspects of the work.

## Conflict of Interest Statement

The authors declare that the research was conducted in the absence of any commercial or financial relationships that could be construed as a potential conflict of interest.
